# Impact of second-line antiretroviral regimens on lipid profiles in an African setting: the DART trial sub-study

**DOI:** 10.1186/1742-6405-11-32

**Published:** 2014-10-02

**Authors:** Zvenyika AR Gomo, James G Hakim, Sarah A Walker, Willard Tinago, Gibson Mandozana, Cissy Kityo, Paula Munderi, Elly Katabira, Andrew Reid, Diana M Gibb, Charles F Gilks, for the DART Team

**Affiliations:** Department of Chemical Pathology, College of Health Sciences, University of Zimbabwe, Avondale, PO Box A178 Harare, Zimbabwe; Department of Medicine, College of Health Sciences, University of Zimbabwe, Avondale, PO Box A178 Harare, Zimbabwe; MRC Clinical Trials Unit, London, UK; Department of Community Medicine, University of Zimbabwe, Harare, Zimbabwe; Joint Clinical Research Centre, Kampala, Uganda; MRC Programme on AIDS/Uganda Virus Research Institute, Entebbe, Uganda; Infectious Disease Institute, Mulago Hospital, Kampala, Uganda; Imperial College, London, UK

**Keywords:** Antiretroviral therapy, Lipid profile changes, Protease inhibitors, Non-nucleoside reverse transcriptase inhibitors, African setting

## Abstract

**Background:**

Increasing numbers of HIV-infected patients in sub-Saharan Africa are exposed to antiretroviral therapy (ART), but there are few data on lipid changes on first-line ART, and even fewer on second-line.

**Methods:**

DART was a randomized trial comparing monitoring strategies in Ugandan/Zimbabwean adults initiating first-line ART and switching to second-line at clinical/immunological failure. We evaluated fasting lipid profiles at second-line initiation and ≥48 weeks subsequently in stored samples from Zimbabwean patients switching before 18 September 2006.

**Results:**

Of 91 patients switched to second-line ART, 65(73%) had fasting samples at switch and ≥48 weeks, 14(15%) died or were lost <48 weeks, 10(11%) interrupted ART for >14 days and 2(2%) had no samples available. 56/65(86%) received ZDV/d4T + 3TC + TDF first-line, 6(9%) ZDV/d4T + 3TC + NVP and 3(5%) ZDV + 3TC with TDF and NVP. Initial second-line regimens were LPV/r + NNRTI in 27(41%), LPV/r + NNRTI + ddI in 33(50%) and LPV/r + TDF + ddI/3TC/ZDV in 6(9%). At second-line initiation median (IQR) TC, LDL-C, HDL-C and TG (mmol/L) were 3.3(2.8-4.0), 1.7(1.3-2.2), 0.7(0.6-0.9) and 1.1(0.8-1.9) respectively. Levels were significantly increased 48 weeks later, by mean (SE) +2.0(0.1), +1.1(0.1), +0.5(0.05) and +0.4(0.2) respectively (p < 0.001; TG p = 0.01). 3% at switch vs 25% 48 weeks later had TC >5.2 mmol/L; 3% vs 25% LDL-C >3.4 mmol/L and 91% vs 41% HDL-C <1.1 mmol/L (p < 0.001). Similar proportions had TG >1.8 mmol/L (0 vs 3%) and TC/HDL-C ≥5 (40% vs 33%) (p > 0.15).

**Conclusion:**

Modest lipid elevations were observed in African patients on predominantly LPV/r + NNRTI-based second-line regimens. Routine lipid monitoring during second-line ART regimens may not be warranted in this setting but individual cardiovascular risk assessment should guide practice.

## Introduction

HIV infection in sub-Saharan Africa and in particular in Central and Southern Africa continues to exact a heavy burden [1]. The past decade has seen a rapid expansion of antiretroviral therapy (ART) rollout particularly in sub-Saharan Africa [1]. This has resulted in a reduction of mortality and morbidity and improvement in quality of life [1].

With an estimated 9.7 million people in lower and middle income countries on ART by the end of 2012 the adverse consequences of treatment are likely to become increasingly significant. Increasing use of ART has been accompanied by the emergence of a range of complications associated with treatment among which short and long-term cardiovascular complications have been observed [2]. Many of these have been poorly documented in the sub-Saharan African setting.

Hyperlipidaemia is a recognised complication of ART which has an impact on the risk of cardiovascular disease [3–8]. The severity of hyperlipidaemia appears to be associated with the type of ART regimen in use. Many ART regimens result in changes in lipid levels, although protease inhibitor based (PI) regimens have been observed to influence lipid changes to a greater degree than other ART regimens [7, 9, 10].

There is a paucity of literature documenting lipid changes in HIV infection and during ART in sub-Saharan Africa. A few reports have, however, began to explore lipid and body-shape changes during ART exposure in this setting [11–15]. These reports predominantly focus on first-line ART regimens including in children. Whilst <3% of those on ART are currently receiving second-line, given the substantial numbers currently on first-line, this will increase significantly over the next decade. There is thus an urgent need for more studies that assess lipid changes during the use of various combinations of antiretroviral drugs in Sub-Saharan African patients taking second-line regimens.

This study aimed to conduct preliminary investigations within the DART Trial (Development of Antiretroviral Therapy in Africa) [16], a randomised trial of routine versus clinically driven laboratory monitoring of antiretroviral therapy in adults with HIV infection in Africa. 3,316 ART-naive adults with CD4 < 200 cells/mm^3^ from Uganda and Zimbabwe initiated first-line ART with Combivir (co-formulated zidovudine + lamuvudine) plus tenofovir (n = 2469), abacavir (n = 300) or nevirapine (n = 547). WHO ART guidelines [17] defined failure triggered criteria to trigger switch to second-line ART (new/recurrent WHO 4 events in all; CD4 < 100 cells/mm^3^ in the 50% randomised to routine laboratory (including CD4) monitoring). In this sub-study we assayed lipids in fasting samples from Zimbabwean participants at switch to second-line ART and ≥48 weeks later. The objective was to determine the magnitude and determinants of lipid changes in patients switched to second-line ART regimens and consider their significance for lipid monitoring recommendations.

## Methods

### Participants

This is an observational prospective sub-study on participants who switched to lopinavir/ritonavir (LPV/r)-containing second-line ART at clinical failure (WHO 4 event) in those randomised to clinically driven monitoring only (CDM); or at clinical or immunological failure (CD4 < 100 cells/mm^3^) in those randomised to laboratory and clinical monitoring (LCM) in the DART trial. Most participants were on LPV/r with a non-nucleoside reverse transcriptase inhibitor (NNRTI) with or without an additional nucleoside reverse transcriptase inhibitor (NRTI) following triple NRTI first-line regimens. Zimbabwean patients were included in the sub-study if they switched to second-line ART before 18 September 2006, allowing 48 weeks follow-up through September 2007, did not interrupt LPV/r-containing ART for >14 days during the first 48 weeks on second-line, and had fasting samples available at switch to second-line and 48 weeks later. Demographic and baseline variables were obtained from the main DART database.

### Determination of lipid profiles

Fasting blood samples were collected into 10 ml EDTA tubes after a 10 hour fast (no intake except for water). Plasma was separated at 3000 g for 10 minutes within an hour of collection. The plasma aliquots were stored at -80°C until analysis was performed. Total cholesterol (TC), low density lipoprotein–cholesterol (LDL-C), high density lipoprotein–cholesterol (HDL-C) and triglycerides (TG) were determined on a Beckman Coulter Synchron CX5 analyzer using appropriate Beckman Coulter reagents (Beckman Coulter, Johannesburg, South Africa).[Synchrone CX].

### Definitions

Body mass index (BMI) was defined by the formula (body weight in Kg)/(height in m)^2^ and categorized as <18.5-underweight, 18.5-24.9-normal, 25.0-29.9-overweight, ≥30.0-obese. To enable comparison with the published literature, criteria from the Third Report of the US National Cholesterol Education Program [18] were used to define abnormal lipid levels as follows: TC ≥5.2 mmol/L, LDL-C ≥3.4 mmol/L, HDL-C ≤1.1, TG ≥1.8 and TC/HDL-C ratio of ≥5 [18]. The Division of AIDS toxicity tables were used to grade lipid toxicity levels [19]. The following abbreviations were used for antiretroviral classes and drugs: nucleoside reverse transcriptase inhibitors (NRTIs), non-nucleoside reverse transcriptase inhibitors (NNRTIs), Protease Inhibitors (PIs), zidovudine (ZDV), lamivudine (3TC), tenofovir (TDF), stavudine (d4T), didanosine (ddI), nevirapine (NVP), efavirenz (EFV), lopinavir/ritonavir (LVP/r).

### Statistical analysis

Plasma lipid levels at switch to second-line ART and 48 weeks subsequently were summarised using medians and interquartile ranges, and compared across groups defined by demographic and other characteristics using the Kruskal-Wallis test. Changes in lipid levels between switch to second-line and 48 weeks were compared using paired t-tests. Multivariable linear regression models were used to identify factors independently associated with changes in lipid levels from switch to ≥48 weeks. All models were adjusted for baseline lipid levels. Analyses were performed using STATA 10 (StataCorp, College Station, TX).

### Ethical approval

The DART trial (ISCRTN13968779) was approved by the Science and Ethics Committee in Uganda, Medical Research Council in Zimbabwe and Imperial College Research Ethics committee in the United Kingdom. Written informed consent was obtained from each enrolled participant.

## Results

Ninety-one DART participants at the Zimbabwe site were switched to second-line ART before 18 September 2006, of whom 65 (71%) had fasting samples at switch and ≥48 weeks later and were included in this sub-study. 26 participants were excluded for the following reasons; 14 (15%) died (no cardiovascular related deaths) or were lost to follow up <48 weeks after switch; 10 (11%) interrupted ART for >14 days; 2 (2%) did not have samples available at both time points. Of the 65 participants included, 34 (52%) were males and 31 (48%) females. The median (IQR) age at switch was 39 (35-44) years, and participants had been on first-line ART for a median (range) 2.2 years (0.9-3.1 years). Characteristics at switch to second-line ART are shown in Table 1. At switch, 16% of participants were underweight according to BMI and an equal proportion, 16%, were overweight or obese. 88% of participants had CD4 counts <100 cells/mm^3^.

**Table 1:** **Demographic data, BMI, CD4 count and plasma lipid levels at initiation of second-line ART (switch from first-line ART)**

At switch to second-line	n (%)	TC mean (se)	^†^P	LDL-C mean (se)	^†^P	HDL-C mean (se)	^†^P	TG mean (se)	^†^P	TC/HDL-C ratio mean (se)	^†^P
All	65	3.4 (0.1)		1.8 (0.1)		0.7 (0.03)		1.4 (0.1)		4.9 (0.2)	
Sex											
Females	31 (48%)	3.8 (0.2)	0.004	2.1 (0.2)	0.006	0.8 (0.04)	0.05	1.3 (0.1)	0.80	4.9 (0.2)	0.66
Males	34 (52%)	3.0 (0.1)		1.5 (0.1)		0.7 (0.04)		1.8 (0.2)		4.8 (0.3)	
Age (years)											
<35	17 (26%)	3.4 (0.2)	0.61	1.7 (0.2)	0.7	0.8 (0.1)	0.78	1.3 (0.3)	0.10	4.8 (0.4)	0.46
35-44	33 (51%)	3.4 (0.2)		1.9 (0.1)		0.7 (0.1)		1.5 (0.1)		4.9 (0.2)	
≥45	15 (23%)	3.2 (0.4)		1.7 (0.2)		0.7 (0.1)		1.5 (0.3)		4.8 (0.3)	
^¥^BMI (kg/m^2^)											
<18.5	10 (16%)	3.0 (0.3)	0.11	1.6 (0.2)	0.04	0.8 (0.1)	0.33	1.0 (0.3)	0.46	4.2 (0.4)	0.45
18.5-24.9	41 (67%)	3.4 (0.1)		1.8 (0.1)		0.7 (0.1)		1.6 (0.2)		4.9 (0.2)	
25-29.9	8 (13%)	3.4 (0.3)		1.8 (0.2)		0.7 (0.1)		1.4 (0.2)		4.8 (0.2)	
30-39.9	2 (3%)	4.0 (0.1)		2.4 (0.1)		0.7 (0.1)		1.7 (0.9)		5.5 (0.5)	
CD4 cell/mm^3^											
<100	57 (88%)	3.3 (0.1)	0.95	1.7 (0.1)	0.47	0.7 (0.02)	0.64	1.5 (0.1)	0.85	4.8 (0.2)	0.73
100-199	6 (9%)	3.9 (0.9)		2.1 (0.6)		0.7 (0.1)		1.4 (0.2)		6.0 (1.0)	
≥200	2 (3%)	4.0 (0.6)		2.2 (0.7)		1.2 (0.1)		0.6 (0.2)		3.5 (0.8)	
ART-during first-line											
ZDV/d4T + 3TC + TDF	56 (86%)	3.4 (0.1)	0.59	1.8 (0.1)	0.30	0.7 (0.03)	0.02	1.5 (0.1)	0.04	5.0 (0.2)	0.09
^±^ZDV/d4T++NVP	9 (14%)	3.4 (0.2)		1.9 (0.2)		0.9 (0.10)		0.9 (0.1)		4.0 (0.4)	

^†^P-values for continuous BMI, Age and CD4 vs lipids calculated using Spearman correlation.

^¥^4 participants with missing weight at initiation of second –line ART.

^±^Including 3 patients who had substituted NVP- > TDF for hepatotoxicity (2) or anti-TB therapy (1) during first-line.

TC: total cholesterol, LDL-C: low density lipoprotein–cholesterol, HDL-C: high density lipoprotein–cholesterol, TG: triglyceride, BMI: Body mass index. All lipid levels are expressed in mmol/L.

### First-line and second-line ART

56 (86%) of the 65 included participants received ZDV/d4T + 3TC + TDF during first-line ART, 6 (9%) ZDV/d4T + 3TC + NVP, and 3(5%) ZDV + 3TC with TDF and NVP at different stages, mostly due to development of TB or NVP-associated rash. Initial second-line regimens were LPV/r + NNRTI in 27(41%), LPV/r + NNRTI + ddI in 33(50%) and LPV/r + TDF + ddI/3TC/ZDV in 6(9%) participants, reflecting the fact that most patients had received 3NRTI first-line. Of the patients on second-line regimens which included an NNRTI, 9/60 (15%) were on EFV and 51/60 (85%) on NVP. During second-line ART, 5 (8%) patients substituted from NVP to EFV; 1 from ddI to d4T, and 1 from EFV to d4T and back to d4T during the course of pregnancy. Only 4 participants had any time off ART during second-line (2 were for 4 days, 1 for 9 days and 1 for 14 days).

### Lipids at switch to second-line ART

At switch to second-line, median (IQR) (mmol/L) plasma fasting lipid concentrations were: TC 3.3 (2.8-4.0), LDL-C 1.7 (1.3-2.2), HDL-C 0.7 (0.6-0.9), TG 1.1 (0.8-1.9), TC/HDL-C ratio 4.6 (4.0-5.6) (Table 1). Using NECP [18] criteria TC was >5.2 mmol/L in 2/65 (3%) participants, LDL-C was >3.4 mmol/L in 2/65 (3%) participants, HDL-C was <1.1 mmol/L in 94% of participants; TC/HDL-C ratio was <5 in 30/65 (60%) participants; and no participant had abnormally raised TG levels.

In the univariate analyses at switch to second-line ART regimens, women had significantly higher TC (+0.8 mmol/L, p = 0.004), LDL-C (+0.6 mmol/L, p = 0.006) and HDL-C (+0.1 mmol/L, p = 0.05). TG levels were not significantly higher (+0.5) mmol/L, p = 0.80 in men at switch to second-line ART regimens. There were no statistically significant differences in lipid levels according to age or CD4 count at switch to second-line ART regimens (Table 1); but LDL-C was higher in those with higher BMI at switch (p = 0.04), with similar non-significant trends for TC and TG.

Patients who had received ART combinations containing NVP in first-line had higher HDL-C (+0.2 mmol/L, p = 0.02) at switch than those who had received ART combinations containing TDF and other NRTIs only (Table 1). Of note there was a statistically significantly higher TC (p = 0.04) in patients who had received ZDV/d4T + 3TC + TDF first-line.

### Anthropometric, immunologic and lipid profile changes from switch to ≥48 weeks of second-line ART

**Figure 1:**
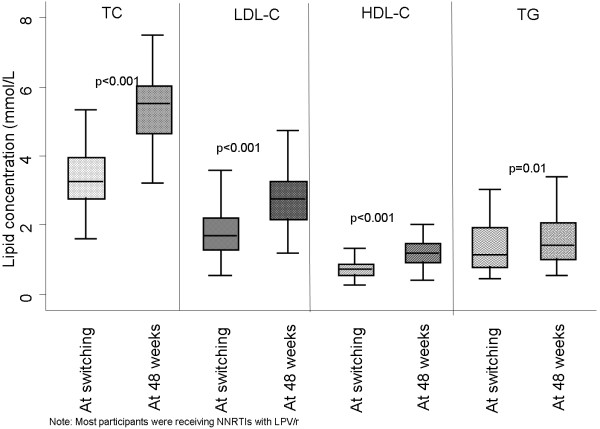
**Fasting plasma lipids at switch to second-line and 48 weeks later.** Footnote 1: Total Cholesterol (TC), Low-density lipoprotein-cholesterol (LDL-C), High-density lipoprotein-cholesterol (HDL-C) and triglycerides (TG) levels were statistically significantly increased ≥48 weeks on switching to second-line ART regimens (p < 0.001).

Fasting lipid profiles (TC, LDL-C, HDL-C, TC/HDL-C ratio and TG) after ≥48 weeks of second-line ART are shown in Table 2; TC, LDL-C and HDL-C remained higher in women persisted, and associations between higher TC, LDL-C and TG and higher BMI strengthened. Lipid levels were significantly increased after ≥48 weeks of second-line ART, by (mean mmol/L) TC +2.0, LDL-C +1.1, HDL-C +0.5 (all p < 0.001) and TG +0.40 (p = 0.01) (Table 3, Figure 1). The percentage increase in lipid levels after ≥48 weeks of second-line ART was TC 59%, LDL-C 61%, HDL-C 71% and TG 29% (Table 3). There was no significant change in TC/HDL-C ratio, p = 0.94 (Table 3).

**Table 2:** **BMI, CD4 Count and lipid levels at ≥48 weeks on second-line ART**

48 weeks after switch to second-line	n (%)	TC mean (se)	^†^P	LDL-C mean (se)	^†^P	HDL-C mean (se)	^†^P	TG mean (se)	^†^P	TC/HDL-C ratio mean (se)	^†^P
All	65	5.4 (0.2)		2.9 (0.1)		1.2 (0.05)		1.9(0.2)		4.9 (0.3)	
Sex											
Females	31 (48%)	5.8 (0.2)	0.03	3.2 (0.2)	0.003	1.3 (0.07)	0.03	1.8 (0.2)	0.71	4.6 (0.2)	0.69
Males	34 (52%)	5.1 (0.2)		2.5 (0.1)		1.1 (0.06)		2.0 (0.2)		5.1 (0.4)	
BMI (kg/m^2^)											
<18.5	4 (6%)	4.8 (0.4)	0.04	2.6 (0.5)	0.05	1.2 (0.3)	0.57	1.0 (0.2)	0.003	4.6 (0.9)	0.55
18.5-24.9	36 (56%)	5.2 (0.2)		2.7 (0.2)		1.2 (0.1)		1.5 (0.1)		5.0 (0.4)	
25-29.9	21 (32%)	5.8 (0.3)		3.1 (0.2)		1.2 (0.1)		2.4 (0.3)		4.8 (0.4)	
30-39.9	4 (6%)	5.9 (0.3)		3.1 (0.4)		1.2 (0.4)		2.8 (1.1)		5.0 (1.0)	
CD4 cell/mm^3^											
<100	2 (3%)	4.6 (0.9)	0.93	2.6 (0.6)	0.28	1.1 (0.1)	0.10	0.8 (0.02)	0.24	4.2 (1.1)	0.31
100-199	10 (15%)	5.7 (0.2)		3.0 (0.2)		1.4 (0.1)		1.8 (0.5)		4.1 (0.4)	
≥200	53 (82%)	5.4 (0.2)		2.8 (0.1)		1.2 (0.1)		1.9 (0.2)		5.1 (0.3)	

TC: total cholesterol, LDL-C: low density lipoprotein–cholesterol, HDL-C: high density lipoprotein–cholesterol, TG: triglyceride, BMI: Body mass index. All lipid levels are expressed in mmol/L.

^†^P-values for continuous BMI and CD4 vs. lipids calculated using Spearman correlation.

**Table 3:** **Plasma Lipid levels changes from switch to ≥48 weeks on second-line ART**

	Initiation of second-line (mean; sd)	48 weeks later (mean; sd)	Changes (SE)	% Change	P-value
	n = 65	n = 65	n = 65		
TC	3.4 (1.0)	5.4 (1.2)	+2.0 (0.1)	59%	<0.001
LDL-C	1.8 (0.7)	2.8 (0.9)	+1.1 (0.1)	61%	<0.001
HDL-C	0.7 (0.2)	1.2 (0.4)	+0.5 (0.05)	71%	<0.001
TG	1.4 (1.0)	1.9 (1.3)	+0.4 (0.2)	29%	0.01
TC/HDLC-C	4.9 (1.4)	4.9 (2.1)	+0.0 (0.3)	0%	0.94

Comparing switch vs ≥48 weeks of second-line ART, the proportion of participants with TC >5.2 mmol/L was 3% vs 60% respectively, and 3% vs 25% respectively had LDL-C >3.4 mmol/L (both p < 0.001). However, as HDL-C also increased, the proportion of participants with HDL-C <1.1 mmol/L decreased after ≥48 weeks of second-line ART from 94% vs 41% (p < 0.001). There were no significant changes in the proportion with TG >1.8 mmol/L (0 vs 3% respectively, p = 0.16) or TC/HDL-C ratio ≥5 (40% vs 33% respectively, p = 0.17). DAIDS toxicity criteria showed a corresponding but small shift to higher grades 3 and 4.

### Factors associated with changes in lipid profiles on second-line

We performed multivariable regression analyses including all demographic and clinical factors weakly (p < 0.20) associated with a change in at least one lipid variable (TC, LDL-C, HDL-C, TG) at ≥48 weeks on univariable analysis, and adjusting for baseline (switch) values (Table 4). All models thus included sex, age, lipid levels at switch to second-line ART, BMI at ≥48 weeks, and type of second-line regimen (EFV vs NVP vs no NNRTI; ddI vs no ddI). Controlling for baseline lipid levels and other factors, in comparison to men, women had significantly greater increases in LDL-C (+0.5, p = 0.04) after ≥48 weeks on second-line ART. Older patients had significantly greater increases in TC (+0.4, p = 0.02), LDL-C (+0.3, p = 0.06) and HDL-C (+0.1, p = 0.02) after ≥48 weeks on second-line ART. There was a trend towards greater TG increases (+0.08 mmol/L, p = 0.07) and smaller HDL-C increases (-0.02 mmol/L, p = 0.08) in those with higher BMI.

**Table 4:** **Multivariable linear regression analyses for factors associated with changes in lipid levels from switch to ≥48 weeks on second-line ART**

At switch to second- line ^*^	n (%) or median (IQR)	TC effect (se)	P	LDL-C effect (se)	P	HDL-Ceffect (se)	P	TG effect (se)	P
Gender									
Females	32 (48%)	+04 (0.3)	0.14	+0.5 (0.2)	0.04	+0.1 (0.08)	0.26	−0.07 (0.3)	0.81
Age (per 10 year increase)	38 (34-43)	+0.4 (0.2)	0.02	+0.3 (0.1)	0.06	+0.1 (0.06)	0.02	−0.04 (0.2)	0.87
BMI (kg/m^2^) per 2 units	21 (17-26)	+0.02 (0.03)	0.47	+0.02 (0.03)	0.41	−0.02 (0.01)	0.08	+0.08 (0.04)^§^	0.07
NVP	50 (77%)	+1.3 (0.4)	0.004	+0.6 (0.3)	0.10	+0.6 (0.2)	<0.001	+0.4 (0.6)	0.43
EFV	9 (14%)	+1.6 (0.5)	0.003	+0.9 (0.4)	0.03	+0.4 (0.2)	0.02	+0.4 (0.6)	0.57
ddI	34 (52%)	−0.04 (0.3)	0.88	−0.1 (02)	0.54	−0.1 (0.1)	0.21	+0.2 (0.3)	0.54

Al l models are adjusted for baseline lipid level.

^§^BMI at week 48 (rather than at switch to second-line) as this was a better predictor.

^*^Reference category: man aged 40 years with BMI 25 at switch, receiving LPV/r plus NRTIs (no NNRTI) without ddI as their second-line regimen.

Patients taking LPV/r with NNRTIs appeared to have greater increases in TC, LDL-C and HDL-C than those taking LPV/r with NRTIs (numbers were however small). Those taking EFV appeared to have larger increases in LDL-C (+0.9 mmol/L, p = 0.03) than HDL-C (+0.4, p = 0.02).

## Discussion

This study reports our observations of lipid changes following switch from first-line ART to second-line regimens containing new classes of antiretroviral drugs with a view to making recommendations on lipid level measurement during second-line ART in an African setting. It should be noted that the DART study (DART) was conducted at a time when triple- nucleoside regimens were in vogue and hence second-line regimens contained a combination of boosted-PIs and NNRTI with or without the addition of NRTIs.

It is well established that increased TC, LDL-C, TC/HDL-C ratio and TG are associated with an increased risk of cardiovascular events, while raised HDL-C reduces this risk [18]. These observations have been made in large studies that indicate these associations to hold across all ethnicities [20]. Observations in resource-rich countries show that HIV infection is associated with low serum lipid levels depending on degree of immunosuppression and viraemia [6, 7]. Several studies in African settings have made similar observations on patients before ART initiation [21–24]. ART has transformed the lives of HIV infected patients but changes in lipid levels are a recognised complication of ART [6, 9, 25]. Although the belief is that Africans in sub-Saharan Africa generally have low lipid levels, there is evidence that this is changing in response to many factors including urbanization with changes in diet, low exercise level and increasing prevalence of hypertension, diabetes and smoking [26].

At baseline (switch to second-line ART after a median of 2.2 years of first-line ART) the TC, LDL-C and TG levels in our study participants were low which is consistent with a low atherogenic risk [18]. The HDL-C levels were however low reflecting a high atherogenic risk. The combination of low TC and low HDL-C resulted in a TC/HDL-C ratio of 4.9 which is reflective of a borderline [18], but relatively high atherogenic risk if one considers the generally low TC and LDL-C levels. After ≥48 weeks of second-line ART predominantly consisting of a combination of PI/r and NNRTI with or without NRTIs there was a marked increase in lipid levels; relative increases of TC 59%, LDL-C 61%, HDL-C 71% and TG 29%. The TC/HDL-C ratio remained unchanged because of the marked increase in HDL-C. Women had a greater increase in LDL-C than men. Older patients had greater increases in TC, LDL-C and HDL-C. Those with higher BMI had greater increases in TG, and smaller increases in HDL-C than those with lower BMI.

In a South African study of 42 patients on an NNRTI-based first-line regimen the TC, LDL-C, HDL-C and TG levels (mean ± standard deviation) following 2.1 years of first-line ART exposure were; 4.75 ± 1.24, 2.65 ± 0.97 and 1.41 ± 0.42 and 1.20 ± 1.18 respectively [13]. In contrast in our patients after 2.2 years of first-line ART (ie at switch to second-line ART); TC, LDL-C and HDL-C levels were lower but TG was higher than in the South African study. The difference could, at least in part, be explained by the lower proportion of women in our study than in the South African study, 48% vs 64% respectively. Indeed there were other important differences between our two studies. Our patients were predominantly on a triple-nucleoside first-line regimen (86%) while the South African patients were all on an NNRTI regimen. However, in a Ugandan study of 374 patients (49% women) who were on a predominantly NNRTI-based first-line regimen (98% on NVP), lipid levels (mmol/L) after 2 years were; 3.91; 2.05; 1.22; 1.35; and 3.4 for TC, LDL-C, HDL-C, TG and TC/HDL-C ratio respectively [14], again TC, LDL-C and HDL-C higher than values at switch to second-line in our Zimbabwean study, but lower than in South Africa. Of note, HDL-C at switch to second-line ART was markedly low in the Zimbabwean study. Interestingly TG levels were more similar in all three studies.

The lipid levels in this Zimbabwean African population are generally low after exposure to a predominantly triple nucleoside first-line ART regimen. The choice of second-line regimens which mostly contained both PI/r and NNRTIs in our study was dictated by first-line exposure. It is therefore not possible to fully ascribe lipid changes to either the PI/r or NNRTI class of antiretroviral drugs, given the small number of patients not receiving NNRTI second-line. PI-based regimens have been associated with elevated lipid levels in several studies [5, 9, 27], although the setting of such changes is often complex [7]. In a study in Benin a significant increase in TC was observed in a cohort of 88 patients who were on an NNRTI first-line regimen [28]. Several studies have shown that NNRTI based regimens are associated with elevated TC and LDL-C levels [8, 9, 28] with regimens combining PIs and NNRTIs showing higher degrees of dyslipidaemia [8]. Various members of the PI class have been shown to have varying effects on lipid changes. In a South African study, after 48 weeks of first-line ART, there was a smaller rise in LDL-C level with an Atazanavir based regimen compared to a Nelfinavir based regimen. Indeed in comparison to Nelfinavir, Atazanvir exposure was not associated with clinically relevant increases in TC, LDL-C or TG. Another limitation of our study is that we did not have a sufficient numbers of patients on EFV versus those on NVP to allow comparison of the impact of these two NNRTIs on lipid change. The percentage increases in TC, HDL-C and TG were high, but because of relatively low baseline levels at switch to second-line ART, the levels attained after exposure to second-line ART were modest.

In this study an almost equal number of men and women of similar ages were recruited which gives us the opportunity to compare lipid changes between men and women unlike studies in resource-rich countries which have predominantly studied men. The fact that women had higher lipid levels than men at switch and 48 weeks later is difficult to explain since normally men have higher athrogenic lipids than women [18]. A partial explanation may be the higher BMI in females compared to males. Indeed both men and women had similar degrees of immunosuppression as shown by similar CD4 counts.

Because of the generally low level of lipids in African populations the facilities to perform lipid assays are limited and expensive. It may therefore not be possible to give a recommendation on routine monitoring of lipids, or even how often lipid levels should be monitored in this population. This preliminary study suggests that lipid changes do occur over the short-to-medium term on second-line LPV/r containing regimens, but are generally mild abnormalities, and are at least partially offset (in terms of cardiovascular risk) by increases in HDL-C, furthermore our results are limited by the short follow-up period. The best approach for the time being may, therefore, be to recommend assessment of total atherogenic risk ie risk factors such as smoking, hypertension, diabetes, BMI and family history to guide monitoring for and indeed treatment of lipids in individual patients. We await further studies to provide more general guidelines on the monitoring and management of lipids during ART based on risk factors for cardiovascular disease, and the impact of various classes of antiretroviral drugs or individual agents.
